# Are we following the guidelines on non-muscle invasive bladder cancer?

**DOI:** 10.1590/S1677-5538.IBJU.2015.0122

**Published:** 2016

**Authors:** Leonardo Oliveira Reis, Juliano Cesar Moro, Luis Fernando Bastos Ribeiro, Brunno Raphael Iamashita Voris, Marcos Vinicius Sadi

**Affiliations:** 1 Divisão de Urologia Oncológica, Faculdade de Medicina, Centro de Ciências da Vida, Pontifícia Universidade Católica de Campinas (PUC-Campinas), Campinas, São Paulo, Brazil;; 2 Disciplina de Urologia, Departamento de Cirurgia da Faculdade de Ciências Médicas da Universidade Estadual de Campinas, (UNICAMP), Campinas, São Paulo, Brazil;; 3 Disciplina de Urologia, Escola Paulista de Medicina (EPM, Unifesp), São Paulo, São Paulo, Brazil

**Keywords:** Urinary Bladder Neoplasms, BCG Vaccine, Practice Guidelines as Topic

## Abstract

**Objectives:**

To evaluate the clinical practice of non-muscle invasive bladder cancer (NMIBC) treatment in Brazil in relation to international guidelines: Sociedade Brasileira de Urologia (SBU), European Association of Urology (EAU) and American Urological Association (AUA).

**Materials and Methods:**

Cross-sectional study using questionnaires about urological practice on treatment of NMIBC during the 32nd Brazilian Congress of Urology. A total of 650 question forms were answered.

**Results:**

There were 73% of complete answers (total of 476 question forms). In total, 246 urologists (51.68%) lived in the southeast region and 310 (65.13%) treat 1 to 3 cases of NMIBC per month. Low risk cancer: Only 35 urologists (7.5%) apply the single intravesical dose of immediate chemotherapy with Mitomicin C recommended by the above guidelines. Adjuvant therapy with BCG 2 to 4 weeks after TUR is used by 167 participants (35.1%) and 271 urologists (56.9%) use only TUR. High risk tumors: 397 urologists (83.4%) use adjuvant therapy, 375 (78.8%) use BCG 2 to 4 weeks after TUR, of which 306 (64.3%) referred the use for at least one year. Intravesical chemotherapy with Mitomicin C (a controversial recommendation) was used by 22 urologists (4.6%). BCG dose raised a lot of discrepancies. Induction doses of 40, 80 and 120mg were referred by 105 (22%), 193 (40.4%) and 54 (11.3%) respectively. Maintenance doses of 40, 80 and 120mg were referred by 190 (48.7%), 144 (37.0%) and 32 (8.2%) urologists, respectively. Schemes of administration were also varied and the one cited by SWOG protocol was the most used: 142 (29.8%).

**Conclusion:**

SBU, EAU and AUA guidelines are partially respected by Brazilian urologists, particularly in low risk tumors. In high risk tumors, concordance rates are comparable to international data. Further studies are necessary to fully understand the reasons of such disagreement.

## INTRODUCTION

Bladder tumor is the seventh most common cancer in males and 17^th^ in females. It affects approximately 110.000 men and 70.000 women every year in the World. During 2012, 38.200 and 17.000 deaths were registered due to BC in Europe and USA, respectively (1, 2). According to data from the National Cancer Institute of Brazil, there were 8,940 new cases in 2014 (6.750 men and 2.190 women in the country (3).

Around 75% of bladder tumors are non-muscle invasive (NMSBC–pTa, pTis or PT1), which evolve differently and need adequate classification and treatment (1-3).

NMIBC treatment is standardized by the Brazilian Society of Urology (SBU), European Association of Urology (EAU) and American Urological Association (AUA). There are specific guidelines that show evidence that their use can improve survival rate of treated patients (4-7). However, daily practice may not correspond to what is recommended, mainly in Brazil, where there are no studies about this subject.

The present study was designed to analyze Brazilian urologist’s ongoing practice in relation to NMIBC and to compare them to SBU, EUA and AUA guidelines (Table-1) in order to appoint differences between theory and practice in Brazilian hospitals and clinics.

**Table 1:** Main International Guidelines on NMIBC.

Classification	EAU guideline	AUA guideline
Low risk	TUR -Single dose of intravesical chemotherapy	TUR -Single dose of intravesical chemotherapy (recommended)
Intermediate risk	TUR -Single dose of intravesical chemotherapy followed by: 1–BCG with maintenance for at least 1 year (level A) 2–Intravesical chemotherapy for 6-12 months (level B)	TUR -Induction with BCG or Mitomicin (recommended) -Maintenance with BCG or Mitomicin (option)
High risk	TUR (re-TUR after 4-6 weeks) -Single dose of intravesical chemotherapy followed by: 1-BCG with maintenance for at least 1 year (level A) -Cystectomy may be considered for patients with high risk of progression (level C) or in cases with failure of BCG treatment (level B)	TUR (re-TUR after 4-6 weeks) -Induction and maintenance with BCG (recommended) -Cystectomy (option)

## MATERIALS AND METHODS

This cross-sectional study was performed during the 32nd Brazilian Congress of Urology. A total of 650 questionnaires were given to urologists and 476 completed them (73%) regarding their experience and actions on NMIBC of low risk (single tumor, <3cm and low histological grade) and high risk (T1 or high grade or carcinoma in situ) submitted to transurethral resection in the last year (4).

Questionnaires contained two parts. First part had questions about demographic data: state where the physician works, local of practice, number of patients attended and treated each month. Second part comprised questions about treatment of NMIBC according to classification (low and high risk), use of intravesical therapy, drug used (chemotherapy, BCG, other), induction and maintenance doses, follow-up, use of new transurethral resection (re-TUR), etc.

All question forms were included in the study, even those incomplete, comprising 476 urologists.

Results were analyzed in terms of percentage of total answered forms.

## RESULTS

Most participants lived in southeast region of Brazil (246 urologists, 51.68%) and 85.3% were from private practice, but not exclusive. 151 urologists worked in teaching facilities (31.7%) and 325 (68.3%) had no relation to teaching institutions (Table-2). In view of the great number of answers regarding simultaneous practice in public, private and teaching institutions, it was not possible to stablish a correlation between adopted treatments and workplace.

**Table 2:** Characteristics of Urological Practice.

WHERE DO YOU WORK?	TOTAL	TOTAL
Private practice	406	85.3%
Private hospital	279	58.6%
Public hospital	163	34.2%
General or community hospital (noteaching)	162	34.0%
University or school hospital	151	31.7%
Cancer treatment center	57	12.0%
Other	31	6.5%

In relation to experience, 310 (65.13%) urologists treated a media of 1 to 3 cases of NMIBC per month (12-36 cases per year), reflecting an adequate experience.

**Figure 1:**
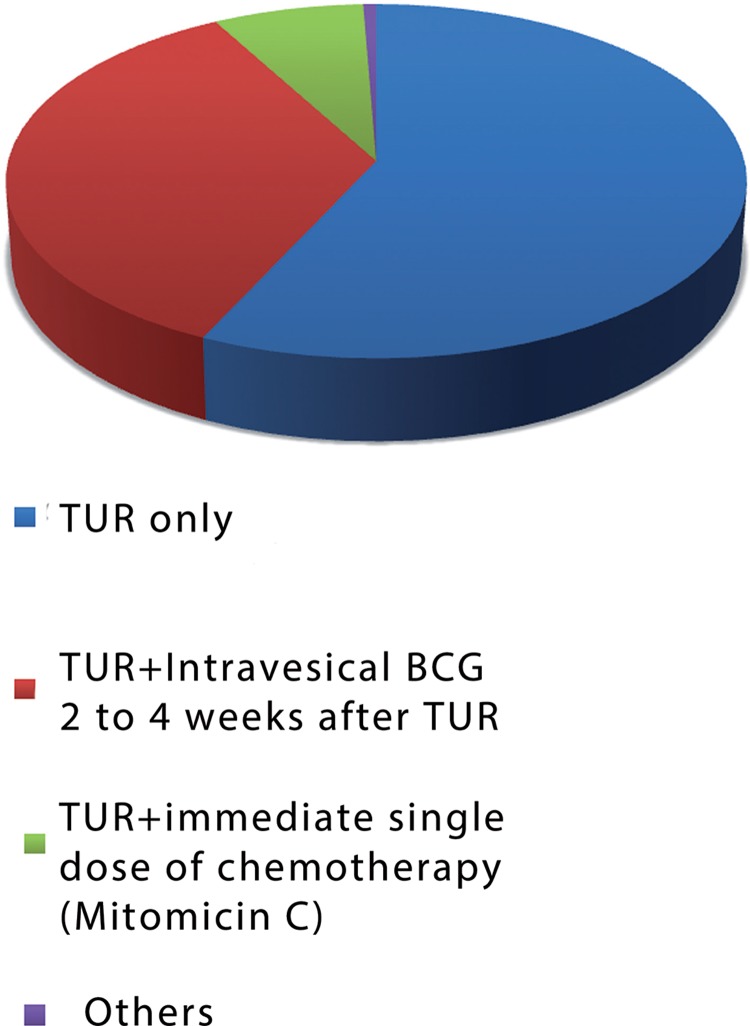
Treatment of low risk tumors.

In relation to treatment of low risk patients, only 35 urologists (7.5%) followed the guidelines of BSU, EAU and AUA, performing TUR followed by a single immediate dose of intravesical chemotherapy with Mitomicin C (4-7); 271 (56.9%) did not use adjuvant therapy, only TUR and 167 (35%) performed TUR+intravesical BCG 2 to 4 weeks after TUR, totaling 91.9% of divergent treatments in relation to guidelines (Figure-1).

Among patients with high risk tumors, 375 (78.8%) urologists performed TUR followed by adjuvant therapy with BCG 2 to 4 weeks after; however, 360 (75.6%) confirmed the use of the maintenance dose. Still, 22 (4.6%) participants performed immediate intravesical chemotherapy with Mitomicin C following TUR, although being a controversial recommendation of the guidelines (4-7) (Table-3).

**Table 3:** Treatment of high risk tumors.

Which is your standard treatment for high risk NMIBC tumors?	Total	Total (%)
TUR+intravesical BCG 2 to 4 weeks after TUR	375	78.8%
Re-TUR	322	67.6%
Radical cystectomy	46	9.7%
TUR+immediate single dose of intravesical chemotherapy (Mitomicin C)	22	4.6%
Neoadjuvant chemotherapy followed by surgical resection	17	3.6%
Only TUR	10	2.1%
Others	6	1.3%

Induction was employed by 300 (63.0%) urologists who used doses of 40, 80 and 120mg (105–22.1%, 193–40.5% and 54–11.3% participants, respectively). Induction time was also heterogeneous: 4 weeks–28 (5.9%), 6 weeks–320 (67.2%) and 8 weeks–101 (21.2%).

Maintenance doses were used by 360 urologists (75.6%). The used dose of 40, 80 and 120mg was employed by 190 (39.9%), 144 (37.8%) and 32 (6.7%) urologists respectively.

In relation to interval, the answers were: weekly during 3 weeks on months 3, 6, 12, 18, 24, 30 and 36 (SWOG protocol)–142 (29.8%); monthly during 1 year– 35 (28.4%); monthly during 2 years–25 (5.3%) (Table-4).

**Table 4:** Maintenance regimens of BCG.

When you use maintenance therapy, how many doses are employed, in which interval and for how long?	Total (N)	Total (%)
Weekly for 3 weeks in months 3, 6, 12, 18, 24, 30 e 36	142	29.8%
Monthly for 1 year	135	28.4%
Monthly for 2 years	25	5.3%
Monthly for 6 months	5	1.1%
Weekly for 3 weeks in months 3.6 and 12	1	0.2%
Twice every 3 or 4 months	1	0.2%
Monthly, continuous	1	0.2%
Weekly for 3 weeks in months 6 and 12	1	0.2%
Weekly for 6 months	1	0.2%
Others	75	15.8%
Did not answered	89	18.7%

These data reveal that, when BCG is used, there is a large variation regarding application regimen and at least 306 participants (64.3%) maintain the treatment for at least one year, according to what is recommended by EAU guidelines for the treatment of high risk bladder tumor. In this respect, there was a 64.3% of accordance rate to the referred guideline.

A new TUR (re-TUR) for high risk patients was performed by 322 participants (67.6%) and 41 (29.6%) did not perform it, reflecting the great number of urologists that do not follow the mentioned guidelines. Among those who performed re-TUR, 220 (46.2%), it was carried out 30 to 45 days following initial TUR, while 71 (14.9%) after 46 to 90 days after.

Most used strains of BCG included Moreau and Connaught (282–59.3% and 125–26.3%, respectively). Besides, the own patient bought the vaccine without reimbursement, according to 204 (42.9%) participants.

In cases where BCG treatment failed, 152 (31.9%) urologists performed a new intravesical treatment with BCG, 115 (24.2%) used intravesical Mitomicin C and 11 (23.3%) performed radical cystectomy (recommended treatment by BSU, EAU and AUA guidelines in that situation). This data points out that 67.8% of participants do not follow the recommendations of the main guidelines for BCG-failed patients (4-7).

## DISCUSSION

Guidelines are important orientations for medical practice, particularly in diseases where the level of evidence is not high enough to avoid questioning. They gather the highest level of evidences so far available to that particular disease. So, it would be reasonable to expect a great level of accordance between medical practice and guidelines. However, in the present study, we observed great disparity between the proposed guidelines (SBU, EAU and AUA) (4-7) and the current practice of Brazilian urologists, according to the questionnaires answers.

Treatment of low risk NMIBC showed the lower compliance of guidelines and only 7.5% of participants followed the recommended treatment (TUR followed by intravesical chemotherapy).

A meta-analysis of 1.476 patients showed a reduction of 11.7% of recurrence rates of BC treated with TUR+immediate instillation of Mitomicin C compared to those treated only with TUR (8). In spite of this benefit, in our study Mitomicin C is rarely used by Brazilian urologists as adjuvant therapy of low risk NMIBC.

Another interesting fact relates to the significant number of participants (35%) that used a dose of BCG 2 weeks after TUR for low risk patients. This practice is still controversial in literature, since meta-analysis showed benefits of BCG therapy in recurrence rates only for patients with high risk tumors (9). However, this is not an absolute fact, since there are at least two other meta-analysis that showed superiority of BCG over Mitomicin C in all high risk groups (10, 11). This blurred fact may explain the high use of BCH for low risk patients in this study.

Our study did not analyze patients with intermediate risk. Witjes et al. (12) observed the use of intravesical chemotherapy in only 29.4% of patients with intermediate risk disease but did not analyze low risk patients.

The treatment of high risk bladder tumors showed a good accordance to the guidelines: 83.4% use adjuvant therapy, 4;6% use Mitomicin C, 78.8% use BCG and among these 75.6% use maintenance doses, and also 64.3% use BCG for at least 1 year, in accordance to the guidelines.

The rate of use of BCG for at least one year in North American and European studies (12-16) varies from 39.8% to 78% in patients with high risk tumors.

An North American study (150 with 494 questionnaires available) showed a 89.7% of use of adjuvant intravesical therapy for high risk tumors, and 87.3% used BCG, reflecting a global use of BCG of 78%. Another epidemiological study from Spain (16) analyzed 2.476 cases of BC being 76.7% NMIBC, with a global rate of BCG use of 39.8%. Anyway, Brazilian rates of compliance of guidelines are comparable to international series.

Isolated TUR was used by 10 participants (2.1%); this number is considerably inferior to that referred by a study performed in European and North American centers, where monotherapy with TUR was used by 9% (12).

Our study detailed the regimen of BCG administration whenever indicated. There was a great variability regarding dose, application interval and total duration of treatment.

According to the guidelines, until now, the evidences show that there is no ideal dose and a reduced one does not lower side effects. However, the distribution of doses probably is based on the concern regarding BCG side effects.

The regimen of BCG use showed a great variation during induction and maintenance. Several regimens were cited by urologists, including never described regimens. However, it is possible to verify that the SWOG protocol was the most used for maintenance (29.8%), what is in accordance to previously described (12, 15).

Guidelines vary in relation to the best regimen to be used; AUA guidelines suggest the use of a standard dose in SWOG regimen (17) for 3 years and EUA guidelines recommend maintenance for at least one year. AUA guidelines are based on a trial with 1.355 patients that used a standard dose and a reduced dose for maintenance during 1 to 3 years with better rates of prevention of recurrence for the 3 year regimen with standard dose, although it was not observed no difference in relation to risk of progression and overall survival (18, 19).

BCG strains are different in relation to phenotype, antigenicity and immunogenic activity, that may influence toxicity, tumor action and clinical efficacy. There are only a few studies that compared effectiveness and collateral effects of different strains, but those used in Brazil are the most common used worldwide (20-22).

Rate of Re-TUR in our study were notably superior in relation to two major international studies (15, 16), where rates varied from 10.4% to 28%. We registered 67.7% of re-TUR.

Following BCG failure, only 23.3% (n=11) indicated cystectomy and not followed international guidelines. Future studies should address criteria for recurrence, failure and intolerance of BCG. Among these, early failure during intravesical treatment reflects a more aggressive disease, justifying precocious cystectomy, while long term recurrence and intolerance are alternatively treated rather than with cystectomy, what could reflect our results.

Our data have never been published and there are very few studies in Brazilian literature. The lack of compliance and use of different regimens other than the European and North American guidelines reaffirm the complexity and diversity of NMIBC treatment (12, 15).

Limitations of the study include the number of urologists that refrained from answering the questionnaire and the fact that it was realized during the Brazilian Congress of Urology, with a potential bias of selection of professionals who tend to recycle and update their knowledge more frequently. Also, regional comparisons were not made, there was no correlation of data with demographics, evaluation of intermediate tumors was not addressed, pathological data were not reviewed and the factors that influenced treatment were not investigated (lack of access to ideal treatment, money issues, patient choice, urologist’s preferences, etc).

## CONCLUSIONS

There is not a significant compliance of Brazilian urologists to BSU, EAU and AUA guidelines, particularly in low risk NMIBC. Further studies are necessary to fully understand the reasons for such discrepancy. The results are similar to those of Europe and USA, but with particular aspects.

## CONCLUSIONS

**SBU =** Sociedade Brasileira de Urologia

**EAU =** European Association of Urology

**AUA =** American Urological Association
